# Extracellular microvesicle microRNAs as predictive biomarkers for targeted therapy in metastastic cutaneous malignant melanoma

**DOI:** 10.1371/journal.pone.0206942

**Published:** 2018-11-06

**Authors:** Fernanda Costa Svedman, Warangkana Lohcharoenkal, Matteo Bottai, Suzanne Egyhazi Brage, Enikö Sonkoly, Johan Hansson, Andor Pivarcsi, Hanna Eriksson

**Affiliations:** 1 Department of Oncology-Pathology, Karolinska Institutet, Stockholm, Sweden; 2 Department of Oncology, Karolinska University Hospital, Stockholm, Sweden; 3 Unit of Dermatology and Venerology, Department of Medicine, Karolinska Institutet, Stockholm, Sweden; 4 Unit of Biostatistics, Institute of Environmental Medicine, Karolinska Institutet, Stockholm, Sweden; 5 Unit of Dermatology, Karolinska University Hospital, Stockholm, Sweden; Rutgers University, UNITED STATES

## Abstract

**Background:**

Mitogen activated-protein kinase pathway inhibitors (MAPKis) improve treatment outcome in patients with disseminated *BRAFV600* mutant cutaneous malignant melanoma (CMM) but responses are of limited duration due to emerging resistance. Although extensive research in mechanisms of resistance is being performed, predictive biomarkers for durable responses are still lacking. We used miRNA qPCR to investigate if different levels of extracellular microvesicle microRNA (EV miRNA) in matched plasma samples collected from patients with metastatic IV *BRAFV600* mutated CMM before, during and after therapy with MAPKis could serve as predictive biomarkers.

**Materials and methods:**

EV miRNAs were extracted from plasma samples from 28 patients collected before and during therapy, measured by quantitative PCR-array and correlated to therapy outcome.

**Results:**

Increased levels of EV let-7g-5p during treatment compared to before treatment (EV let-7g-5p_delta) were associated with better disease control with MAPKis (odds ratio 8568.4, 95% CI = 4.8–1.5e+07, P = 0.000036). Elevated levels of EV miR-497-5p during therapy were associated with prolonged progression free survival (PFS) (hazard ratio = 0.27, 95% CI = 0.13–0.52, P <0.000061).

**Conclusions:**

EV miRNAs let-7g-5p and miR-497-5p were identified as putative novel predictive biomarkers of MAPKi treatment benefit in metastatic CMM patients highlighting the potential relevance of assessing EV miRNA during and after treatment to unravel novel mechanisms of resistance.

## Introduction

There has been a rapid development of novel treatments for metastatic cutaneous malignant melanoma (CMM) [1–6]. Approximately 50% of all CMMs harbor a mutation in *BRAF*, predominantly a substitution of valine to glutamine in codon 600 (*BRAFV600E*), conferring a constitutive activation of the mitogen activated-protein kinase (MAPK) pathway [7]. MAPK pathway inhibitors (MAPKis), including BRAF- and MEK-inhibitors (BRAFis; MEKis), induce rapid responses and improved survival in a majority of patients with *BRAFV600* mutant disseminated CMMs as compared to conventional chemotherapy [2–4, 8–10]. Resistance and disease progression frequently occur within approximately 7 months with single agent and between 11–12 months with combined treatment [8–10]. There is thus a need to identify biomarkers for predicting durable responses to MAPKis in advanced CMM.

Assessing tumor biomarkers in blood samples, i.e. “liquid biopsies”, would be preferable to tissue biopsies, as blood sampling is less invasive and may better capture global tumor heterogeneity compared to a single site biopsy. Extracellular microvesicles (EVs) are released by cells and classified into exosomes, microvesicles or apoptotic bodies depending on their size, which ranges between 40 and 5000 nanometers in diameter [11–12]. EVs are composed of a lipid membrane and may contain virtually all the molecular constituents of a cell such as proteins, lipids, microRNAs (miRNAs), mRNAs and DNA [11–12]. EV miRNAs have been reported to participate in intercellular communication and to serve as molecular signatures in cancer [11–13].

At disease progression or during treatment, CMMs release short, non-coding RNAs or miRNAs within circulating EVs, which may serve as diagnostic, prognostic or predictive biomarkers [14–17]. The role of circulating EV miRNAs remains unclear. In cells, miRNAs regulate gene expression at the post-transcriptional level and are aberrantly expressed in different cancers [18–19]. In CMM patients, circulating miRNA signatures have been correlated to tumor burden, recurrence and overall survival (OS) [15, 17]. Deregulated expression of specific miRNAs, such as overexpression of miR-200c, has been associated with increased sensitivity to BRAFi and MEKi in melanoma cell lines [20]. In several cancers including CMM, EV miRNAs have emerged as promising biomarkers since they are highly resistant to degradation and easily accessible [11–12, 21].

To our knowledge, no previous reports have investigated the correlation between EV miRNA levels in plasma and outcome in patients with disseminated CMM receiving MAPKis. To address this, we have analyzed EV miRNAs in sequential plasma samples from metastatic CMM patients treated with BRAFis alone or in combination with MEKis.

## Materials and methods

### Patients and plasma samples

Plasma samples and clinical data were collected from 28 metastatic CMM patients before and during treatment with MAPKis between March 2012 and May 2015 at the Department of Oncology, Karolinska University Hospital, Sweden. After centrifugation, the plasma samples were stored in -70 degrees Celsius until the analysis. The study was conducted in accordance with Good Clinical Practice/the Declaration of Helsinki with informed consent from all patients and was approved by the Stockholm Regional Ethics Committee, Karolinska Institutet, Sweden.

The clinical information included age at treatment start, sex, stage as well as metastatic classification (M-class) at treatment start, according to the 2009 American Joint Committee on Cancer (AJCC) CMM staging [22], lactate dehydrogenase (LDH) level before and during therapy (S1 Table), type and line of MAPKi treatment, response to treatment, date of disease progression and/or death (Table 1). Elevated LDH is typically seen in patients with high tumour burden and high proliferation and is still used to classify the extension of metastatic disease [22]. It is an important prognostic factor and high LDH values are correlated with worse prognosis [22]. LDH values >4.3 mKat/L were considered elevated according to the upper reference level at the Karolinska University Laboratory.

**Table 1:** Clinical and outcome data from all 28 patients with metastatic cutaneous malignant melanoma patients treated with MAPK inhibitors.

Patient	Sex	Age (years)	Metastatic classification	Treatment	Line of treatment	Treatment response	PFS^1^ (days)
1^5^	M	60	M1c^8^	Dabrafenib	First	DC^2^	186
2	F	50	M1c	Vemurafenib	First	DC	199
3	M	32	M1c	Vemurafenib	First	DC	148
4^5^	M	52	M1c	Dabrafenib/Trametinib	First	DC	282
5	F	44	M1c	Vemurafenib	First	DC	118
6	M	73	M1c	Vemurafenib	First	NE^3^	35
7	F	39	M1c	Vemurafenib	First	DC	80
8	M	72	M1a^6^	Vemurafenib	First	DC	627
9	M	50	M1c	Vemurafenib	First	NR^4^	35
10	F	65	M1c	Vemurafenib	First	DC	328
11	M	48	M1c	Vemurafenib	First	NR	98
12	F	56	M1c	Vemurafenib	First	DC	224
13	M	80	M1c	Vemurafenib	Second	NR	29
14	M	60	M1c	Vemurafenib	First	DC	245
15	M	42	M1c	Vemurafenib	First	NR	124
16^5^	F	43	M1b^7^	Encorafenib/Binimetinib	First	DC	506
17	M	41	M1c	Dabrafenib	First	NR	83
18	M	52	M1c	Dabrafenib/Trametinib	First	NR	70
19	M	61	M1c	Dabrafenib/Trametinib	First	DC	231
20	M	68	M1c	Dabrafenib/Trametinib	First	DC	335
21^5^	F	57	M1c	Encorafenib/Binimetinib	First	DC	387
22	F	60	M1c	Dabrafenib/Trametinib	First	DC	199
23	F	48	M1c	Vemurafenib/Cobimetinib	First	DC	159
24^5^	F	69	M1c	Encorafenib	First	DC	169
25	M	69	M1c	Dabrafenib	First	NR	91
26	F	69	M1c	Dabrafenib/Trametinib	First	DC	266
27	F	64	M1c	Dabrafenib/Trametinib	First	DC	242
28	M	71	M1c	Dabrafenib	First	NE	18

^1^ PFS: Progression-free survival

^2^ DC: disease control

^3^NE: Not evaluable

^4^NR: Non-responder

^5^Treatment within clinical trials

^6^ M1a: distant metastases to the skin, subcutis and extra-regional lymph nodes with normal LDH

^7^ M1b: distant metastases to the lungs with normal LDH

^**8**^ M1c: distant metastases to all other visceral sites or distant metastases to any site associated with elevated LDH

Pre-treatment plasma samples were available from all 28 patients, matched plasma samples during treatment were collected from 25/28 patients (two patients died before during treatment samples were collected and one was missed) and plasma samples after disease progression were collected from 8 of the 28 patients. The pre-treatment samples were collected 0–13 days before treatment start, whereas the number of days between the start of therapy and the during treatment sample collection ranged between 10–57 days (median of 22 days).

Twenty-six patients had M1c CMM (93%) and one patient each had M1a and M1b disease (Table 1). All patients except one were treated with MAPKis as first-line therapy. One patient received ipilimumab first followed by MAPKi (Table 1). The MAPKi agents used, alone or in combination, are described in Table 1. Cytotoxic T lymphocyte-associated antigen 4 (CTLA-4) is a protein inhibiting early T cell activation. Ipilumumab is a monoclonal CTLA-4 antibody that blocks the interaction of CTLA-4 with its ligands and thus increases immune response and anti-tumor immunity. It is presently not known whether pretreatment with ipilumumab will affect responsiveness to MAPKi treatment.

Twenty-three patients were treated outside of clinical trials according to the standard local follow-up scheduled every fourth week and with radiological evaluation performed every 8–12 weeks. Therapy response was based on clinical/radiological investigations evaluated by a team including oncologists, radiologists and pathologists. Computed tomography (CT), magnetic resonance imaging and/or positron emission CT tomography were used. Four patients received therapy within clinical trials (three in Columbus/NCT 01909453, one in Combi D/NCT01584648) and one patient in a Compassionate Use Program for dabrafenib. These patients were followed according to the protocols and were evaluated with imaging according to RECIST 1.1 [23].

Patients considered as having reached DC included patients with stable disease (SD) or with a decreased number and/or size of the existing metastases, without appearance of new lesions. NRs were defined as having lesions with increasing size or new lesions shown by imaging and/or clinical examination without any previous response to the therapy. In case of SD or “mixed response” (i.e. increased size of some lesions and decreased size of others) a confirmatory imaging was performed within 6–8 weeks. A patient was classified as NR if the scan confirmed progression and this date was recorded as date of progression. The PFS was calculated from treatment start until the date of confirmed progression or the date of death from any cause.

### Preparation of plasma-derived EVs and isolation of RNA

MiRCURY Exosome Isolation Kit-Serum and Plasma (Exiqon, Denmark) was used for isolation of plasmatic EVs. Briefly, 600 μl of plasma was treated with thrombin for 5 minutes at room temperature to remove clotting factors. The supernatant was mixed with 200 μl of precipitation buffer and incubated at 4°C for overnight and centrifuged (10,000 g for 30 min at room temperature). The pellet was vortexed for 5–15 minutes in 270 μl resuspension buffer until completely re-suspended. The EV miRNA was extracted using miRCURY RNA isolation kit-biofluids (Exiqon, Denmark) according to the manufacturer´s instruction. RNA spike-in template mixture (Exiqon, Denmark) was added as a quality control of the downstream PCR analysis. dx.doi.org/10.17504/protocols.io.tuienue [PROTOCOL DOI]

### MiRNA expression profiling

MiRNA expression profiling was performed using miRCURY LNA Universal RT miRNA PCR Human panel I (Exiqon, Denmark) covering 372 human miRNAs. All miRNAs were polyadenylated and reverse transcribed in cDNA in a single reaction step. RNA isolation cDNA synthesis efficiency was examined by the detection of spike-in UniSp2, UniSp4 and UniSp6 to ensure that the quality of the input RNA was sufficiently high for effective amplification. cDNA and Exilent SYBR Green master mix (Exiqon, Denmark) were transferred to qPCR panels preloaded with primers. Amplification was performed in a Lightcycler 480 (Roche). Normalization was performed according to Exiqon guidelines based on the average of the assays detected in all samples. For the present study, this included 24 assays. The stability of the average of 24 microRNAs is higher than any single microRNA in the data set as measured by the NormFinder software. The following formula was used to calculate the normalized Cq: Normalized Cq = average Cq (n)–assay Cq (sample) A higher value thus indicates that the microRNA is more abundant in the particular sample. (S1 Table).

### Recurrence samples analysis by qPCR

Plasma samples after tumor recurrence together with matched samples before and during treatment were subjected to EV isolation and RNA extraction. The levels of let-7g-5p and miR-497-5p were determined by qPCR with Megaplex poolTM with pre-amplification (Life Technologies, CA, USA) according to the manufacturer’s instruction. Cel-miR-39 was used as a spike-in control.

### Data analysis

Raw Cq values and melting points detected by the Lightcycler software were exported. Reactions with several melting points or with melting points that were not within the assay specification were flagged and removed from the data set. An evaluation of the negative control was performed by removing the reactions giving Cq values that were within 5 Cq values of the negative control reaction. For assays that did not yield any signal on the negative control, the upper limit of detection was set to Cq = 37. Hemolysis assessment was performed by the detection of miR-451, a miRNA highly expressed in red blood cells and miR-23a which is unaffected by hemolysis. The data was considered as affected by hemolysis if delta Cq between these 2 miRNAs was more than 8.

### Statistical analysis

The probability of responding (DC versus NR) was analyzed by logistic regression. The area under the curve was computed and reported for the response to treatment analysis. We evaluated the following variables: baseline age, sex, LDH and all the available miRNAs. For each miRNA, the values before and during treatment were considered as well as the difference between these values (delta miRNA). Patients that died within the follow-up time were censored. We analyzed time to progression with Cox regression analysis correlating the levels of EV miRNA in plasma samples collected before and during treatment, and the delta-miRNA levels with the risk for progression or death, whatever came first. A multivariate analysis was performed for the clinical variables, but was not performed for the miRNA Cox analysis considering the power limitations. Violations of the proportionality of the hazards function across covariate patterns were tested with Schoenfeld’s residuals. Survival probabilities were estimated with the Kaplan-Meier methods.

Log-rank test was used for marginal differences between groups. Estimates were considered only when based on at least 10 valid non-missing observations. P-values less than 0.05 were considered statistically significant. The significance was adjusted for the multiple testing with the Benjamini–Hochberg (BH) procedure controlling the false discovery rate at level 0.05 [24].

## Results

### Patient characteristics

Overall, 19/28 patients (68%) were classified as having disease control (DC) and 7/28 patients (25%) as non-responders (NRs) (Table 1). Response could not be evaluated in two patients (7%) because of death before the first response evaluation (Table 1). Among patients with DC, only 37% (7/19 patients) were men whereas all NRs were males (Table 1). The median age was 59 years in patients with DC (range 32–72 years) and in NRs 50 years (range 41–80 years) (Table 1). The progression-free survival (PFS) ranged between 18 and 627 days, with a median of 177.5 days (Table 1). Nineteen out of 28 patients (68%) had elevated pre-treatment LDH levels (S2 Table).

There was no correlation between sex or age and outcome (treatment response or PFS) in the univariate analyses. Elevated LDH levels before treatment were associated with a shorter PFS (hazard ratio (HR) = 2.5, 95% CI = 1.4, −4.3, P = 0.003). The association between elevated LDH and shorter PFS remained significant after adjusting for sex and age (HR = 2.1, 95% CI = 1.03–4.2, P = 0.04). No correlation between LDH and therapy response was found.

### Characterization of plasma-derived EV

Before study start, four serum samples from healthy donors were analyzed and EVs were identified by using transmission electron microscope and by western blot analysis based on the expression of the exosomal marker CD63 as part of the optimization of the methods (S1 Fig).

### Changes in plasma-derived EV miRNA levels predict response to MAPKi therapy

**Fig 1:**
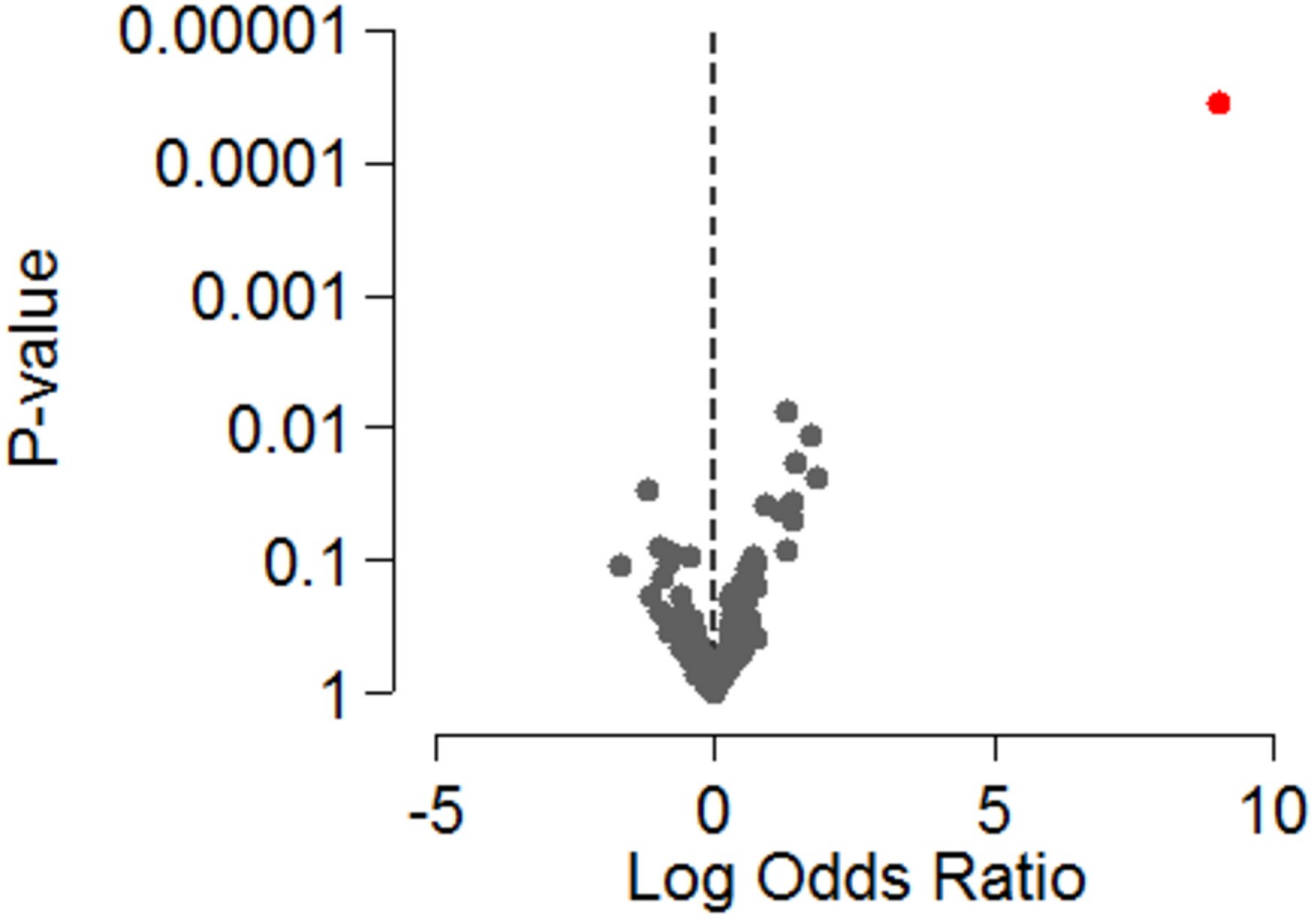
Volcano plot depicting log odds ratios and *P*-values of the extracellular microvesicle (EV) microRNA delta_levels* in plasma and response to MAPK-inhibitors in 25 patients with metastatic cutaneous malignant melanoma. The let7g-5p is specified in red. * The difference in EV miRNA levels during and before treatment.

**Fig 2:**
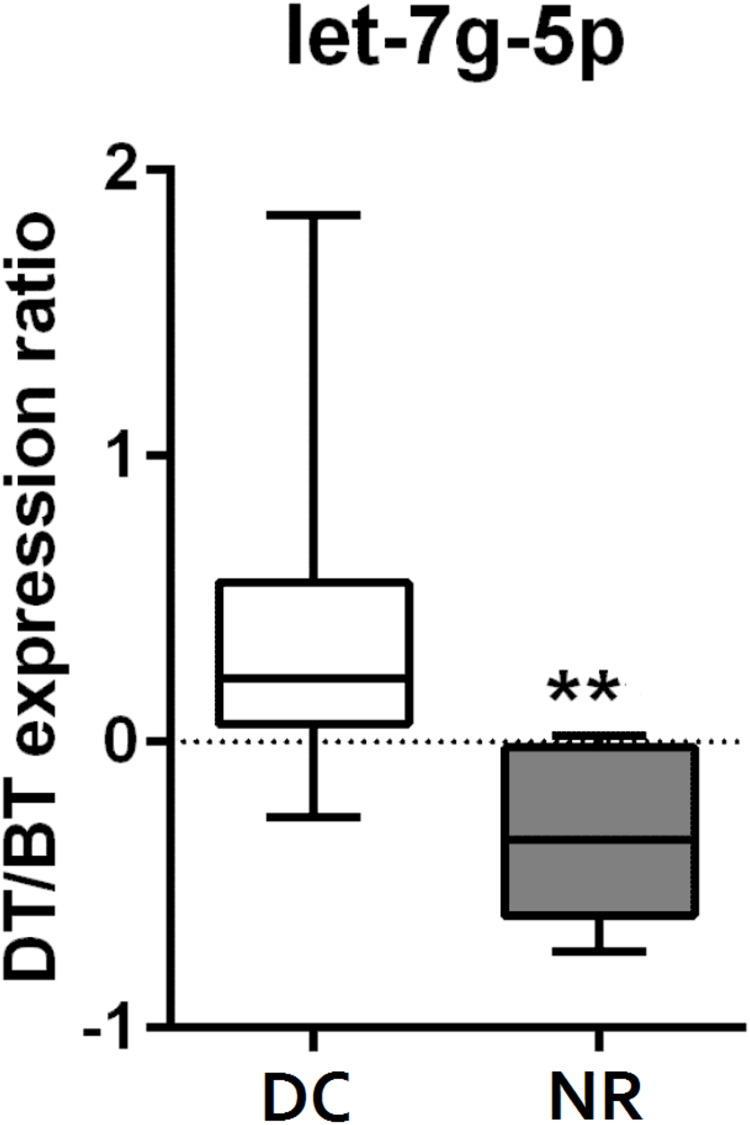
Box plot showing extracellular microvesicle (EV) let-7g-5p delta_levels* in plasma from 25 patients with metastatic cutaneous malignant melanoma, with disease control (DC) and non-responders (NR), treated with MAPK-inhibitors. * The difference in EV let-7g-5p levels before (BT) and during treatment (DT) ** *P* < 0.001, Mann-Whitney U-test.

The logistic regression analysis did not demonstrate a significant association between EV miRNA levels and response to therapy with MAPKi (DC versus NR) either before or during treatment in the 25 patients with matched plasma samples (S3 and S4 Tables). In the logistic regression analysis, patients with increased levels of EV let-7g-5p during treatment compared to before treatment (EV let-7g-5p_delta) had a higher likelihood of response to MAPKis (odds ratio (OR) 8568.4, 95% CI = 4.8–1.5e+07, *P* = 0.000036). The association was significant after controlling for a false discovery rate of 5% in multiple testing (Table 2, Figs 1 and 2). We examined the distribution plots for the levels of EV let7g-5p in our cohort to better understand the high OR and large CI for EV let-7g-5p. We found one outlier sample, which could be a result of some artifact. Excluding this patient had no effect in the results that were identical in both analyses.

**Table 2:** Increased levels of extracellular microvesicular (EV) let-7g-5p_delta^1^ correlates with response to MAPK inhibitors in 25 patients with metastatic cutaneous malignant melanoma treated with MAPK inhibitors.

EV microRNAs^2^	Odds Ratio	Confidence interval	*P*-value	Significance in the Benjamini–Hochberg
hsa-let7g-5p	8568.35	4.82–1.5e+07	0.00003	Yes
hsa-mir-30b-5p	3.72	1.04–13.20	0.00763	No
hsa-mir-126-3p	5.65	1.03–30.87	0.01193	No
hsa-let7c-5p	4.34	0.96–19.62	0.01870	No
hsa-mir-137	6.27	0.46–84.36	0.02451	No
hsa-mir-130a-3p	0.29	0.08–1.10	0.02962	No
hsa-mir-145-5p	4.16	0.86–19.99	0.03752	No
hsa-let7d-5p	2.56	0.90–7.26	0.03939	No
hsa-mir150-5p	3.23	0.83–12.47	0.043071	No
hsa-let7a-5p	4.00	0.76–20.85	0.050306	No

^1^ The difference in EV let-7g-5p levels before and during treatment.

^2^ The 10 most significant EV microRNAs detectable in at least 10 patients, based on the *P-*value are listed in Table 2.

**Fig 3:**
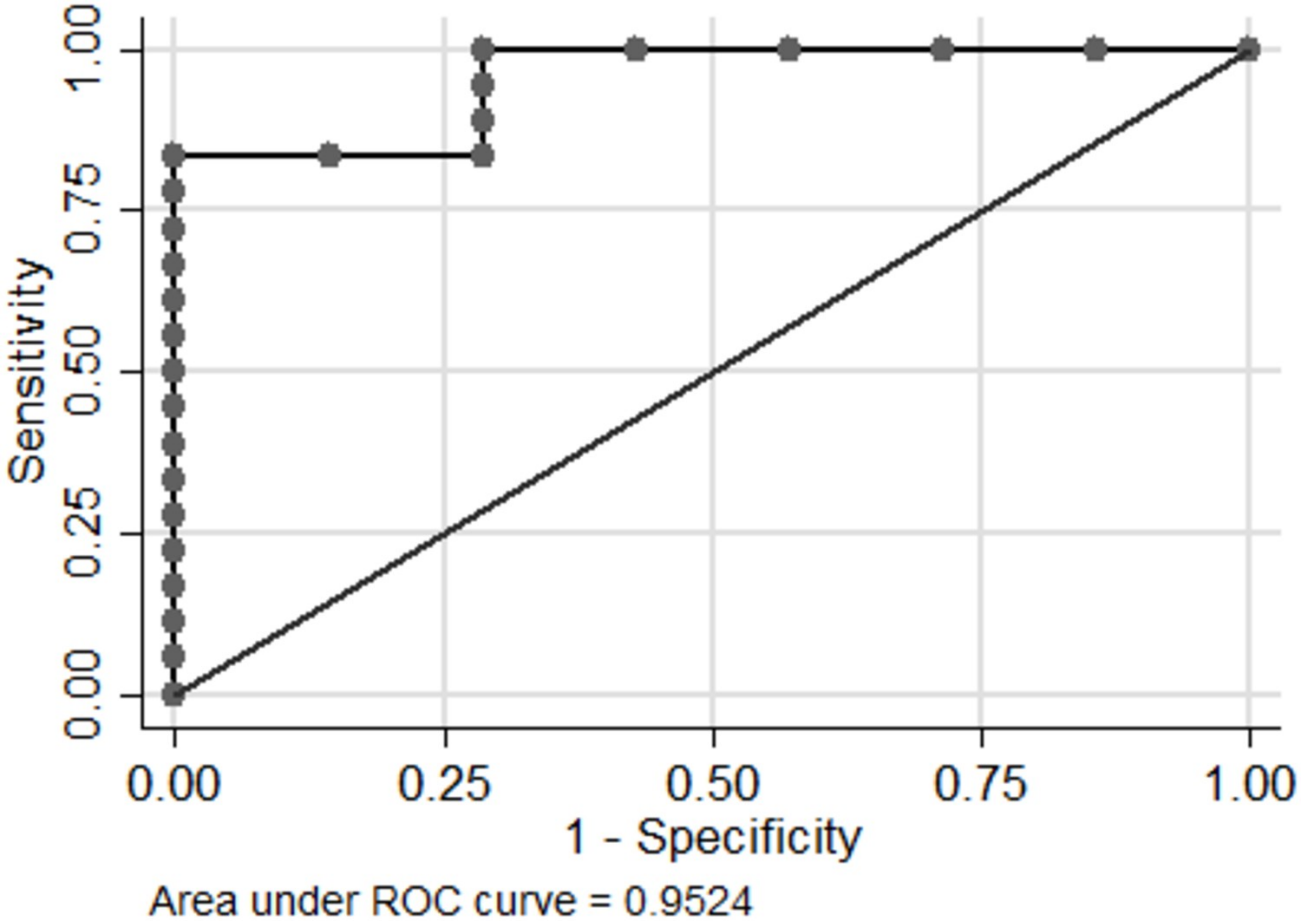
Receiver operating characteristic (ROC) curve generated from the median difference of extracellular microvesicle (EV) let-7g-5p delta_levels* in plasma and response to MAPK-inhibiting treatment in 25 patients with metastastic cutaneous malignant melanoma. The diagonal represents the line of no-discrimination. Points above this line indicate a good classification results (better than random) and below this line a poor classification results (worse than random). The area under the ROC curve is an estimate of the overall accuracy of the test, a value of 0.9524 indicates excellent accuracy. * The difference in EV let-7g-5p levels before (BT) and during treatment (DT).

The receiver operating characteristic (ROC) curve analysis for therapy response demonstrated that the area under the curve (AUC) significantly separated increased levels of EV let-7g-5p during therapy compared to before treatment (EV let-7g-5p_delta), in patients with DC from decreased EV let-7g-5p_delta levels in NRs to MAPKis (AUC = 0.95, P = 0.001) (Fig 3). For the cutoff value of 0.11, the specificity and sensitivity of EV let-7g-5p_delta change was 100% and 72% in patients with DC vs. NRs, respectively (Fig 3).

The qPCR analysis of EV miRNAs in plasma samples collected from 8 patients before and during treatment and at disease progression showed no significant difference in EV let7g-5p levels between patients with DC and NRs (S5 Table).

### Levels of plasma-derived EV miR-497-5p during treatment predict PFS

**Fig 4:**
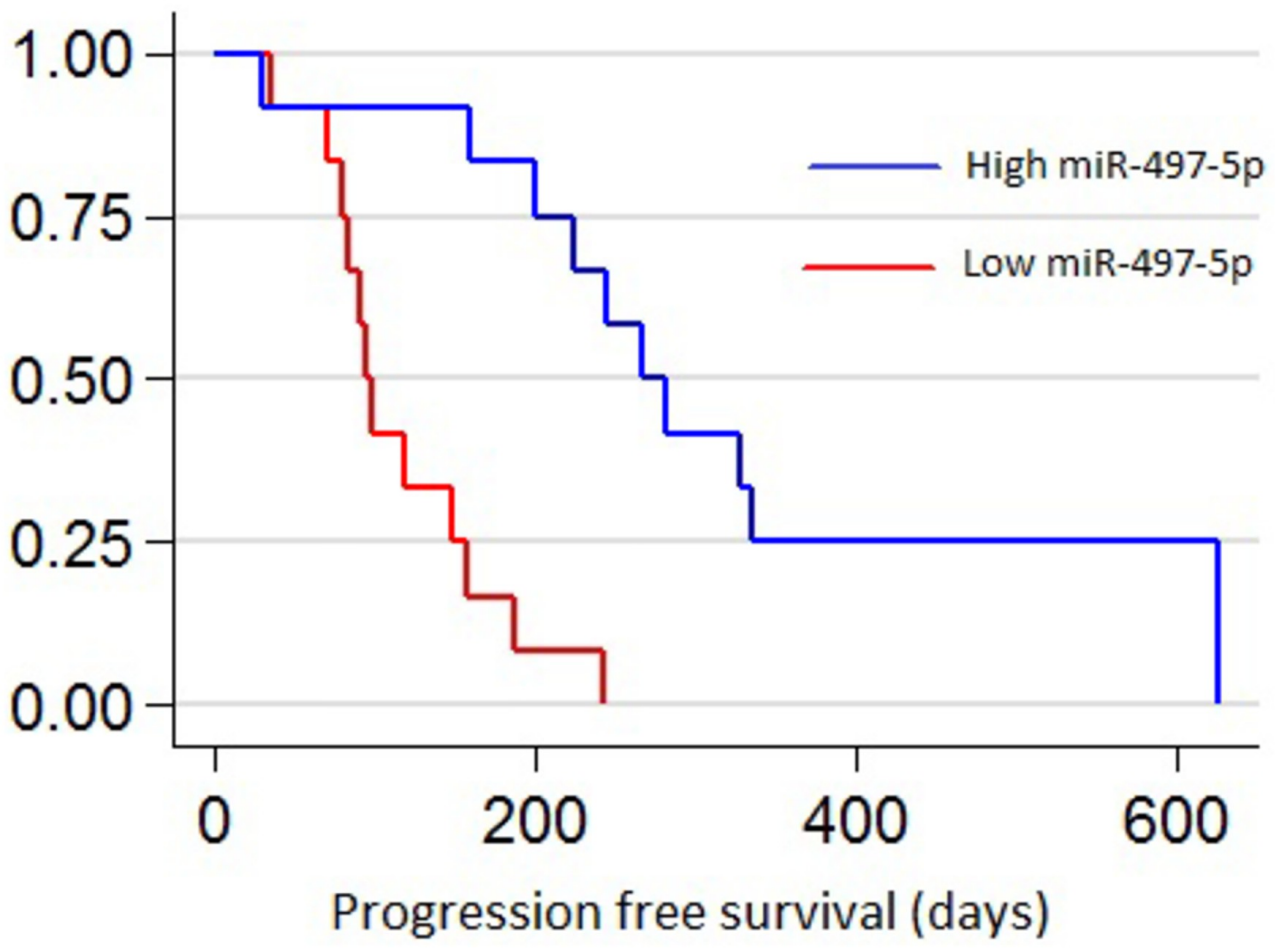
Kaplan-Meier analysis demonstrating the correlation between extracellular microvesicule (EV) miR-497-5p levels in plasma during treatment (DT) with MAPK-inhibitors and progression free survival (PFS) in 25 patients with metastatic cutaneous malignant melanoma.

The Kaplan-Meier analysis of EV miRNA levels during treatment in the 25 patients treated with MAPKis revealed that higher levels of EV miR-497-5p were significantly correlated with prolonged PFS (*P* = 0.0001) (Fig 4). The group with high levels of EV miR-497-5p (high Cq > -4.66) had a median PFS of 274 days compared to 133 days in the group with low levels of EV miR-497-5p (low Cq < -4.66).

**Fig 5:**
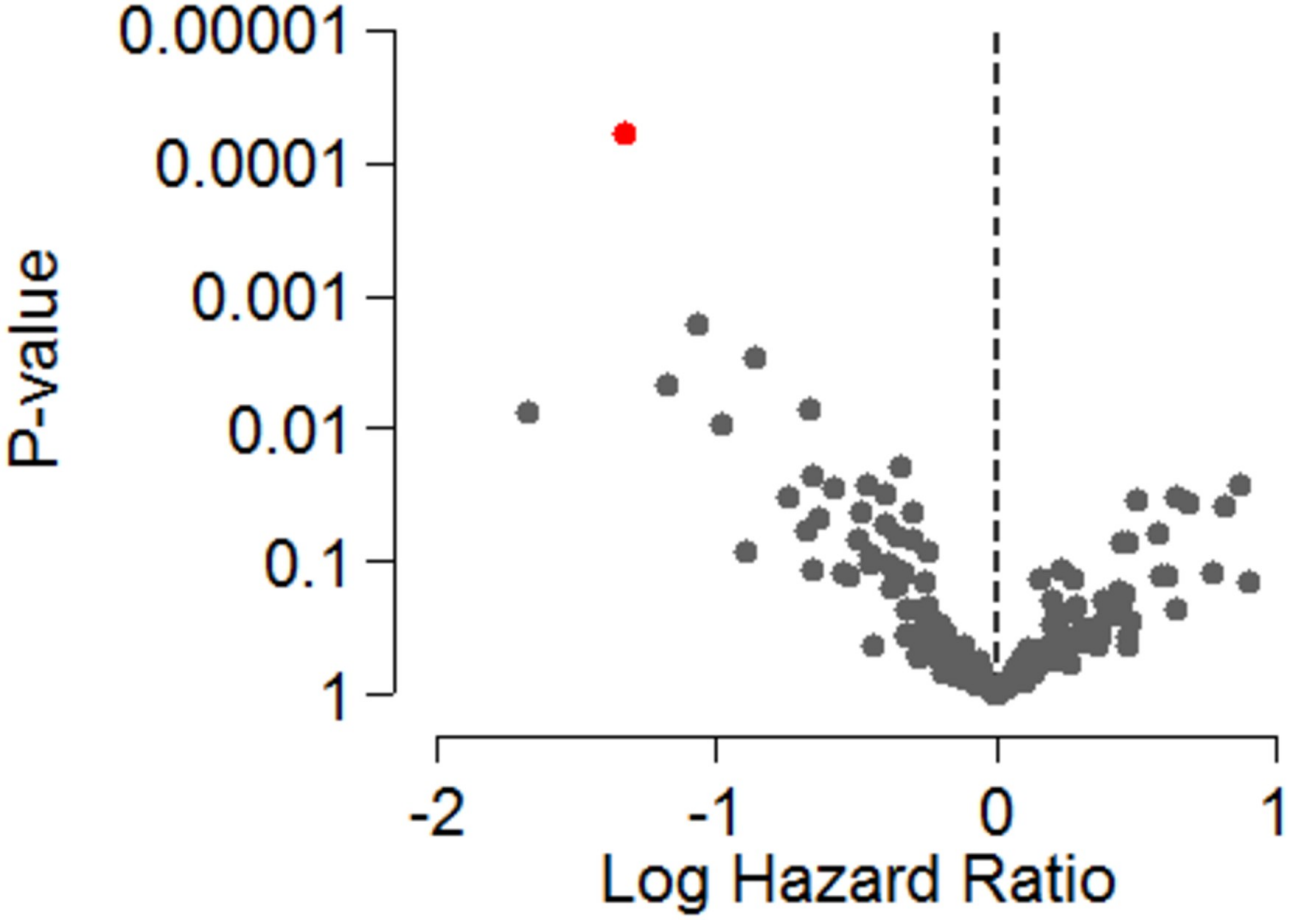
Volcano plot showing log hazard ratios and *P*-values of extracellular microvesicle (EV) microRNA levels in plasma during treatment with MAPK-inhibitors in 25 patients with metastatic cutaneous malignant melanoma. The miR-497-5p is highlighted in red.

In the Cox proportional regression analysis, elevated levels of EV miR-497-5p in plasma during therapy were significantly correlated with reduced risk of disease progression (HR = 0.27, 95% CI = 0.13–0.52, P < 0.0001) (Table 3, Fig 5). There was no significant correlation between EV miRNAs levels and PFS in samples collected before treatment or in delta values (S6 and S7 Tables).

**Table 3:** Increased levels of mir-497-5p during treatment correlates with prolonged progression free survival in 26 patients with metastatic cutaneous malignant melanoma treated with MAPK inhibitors.

EV microRNAs^1^	Hazard Ratio	Confidence interval	*P*-value	Significance in the Benjamini–Hochberg
hsa-mir-497-5p	0.26	0.13–0.52	0.00006	Yes
hsa-mir-150-5p	0.34	0.16–0.72	0.00168	No
hsa-mir-125a-5p	0.42	0.21–0.84	0.00295	No
hsa-mir-125b-5p	0.30	0.13–0.71	0.00481	No
hsa-mir-205-5p	0.51	0.31–0.85	0.00719	No
hsa-let7g-5p	0.18	0.04–0.73	0.00780	No
hsa-mir-126-5p	_ 0.37	0.17–0.79	0.00934	No
hsa-mir-375	0.70	0.54–0.92	0.01977	No
hsa-mir-26a-5p	0.51	0.30–0.88	0.02298	No
hsa-mir-423-5p	2.39	1.12–5.08	0.02718	No

^1^ The 10 most significant EV microRNAs detectable in at least 10 patients, based on the *P-*value are listed in Table 3.

**Fig 6:**
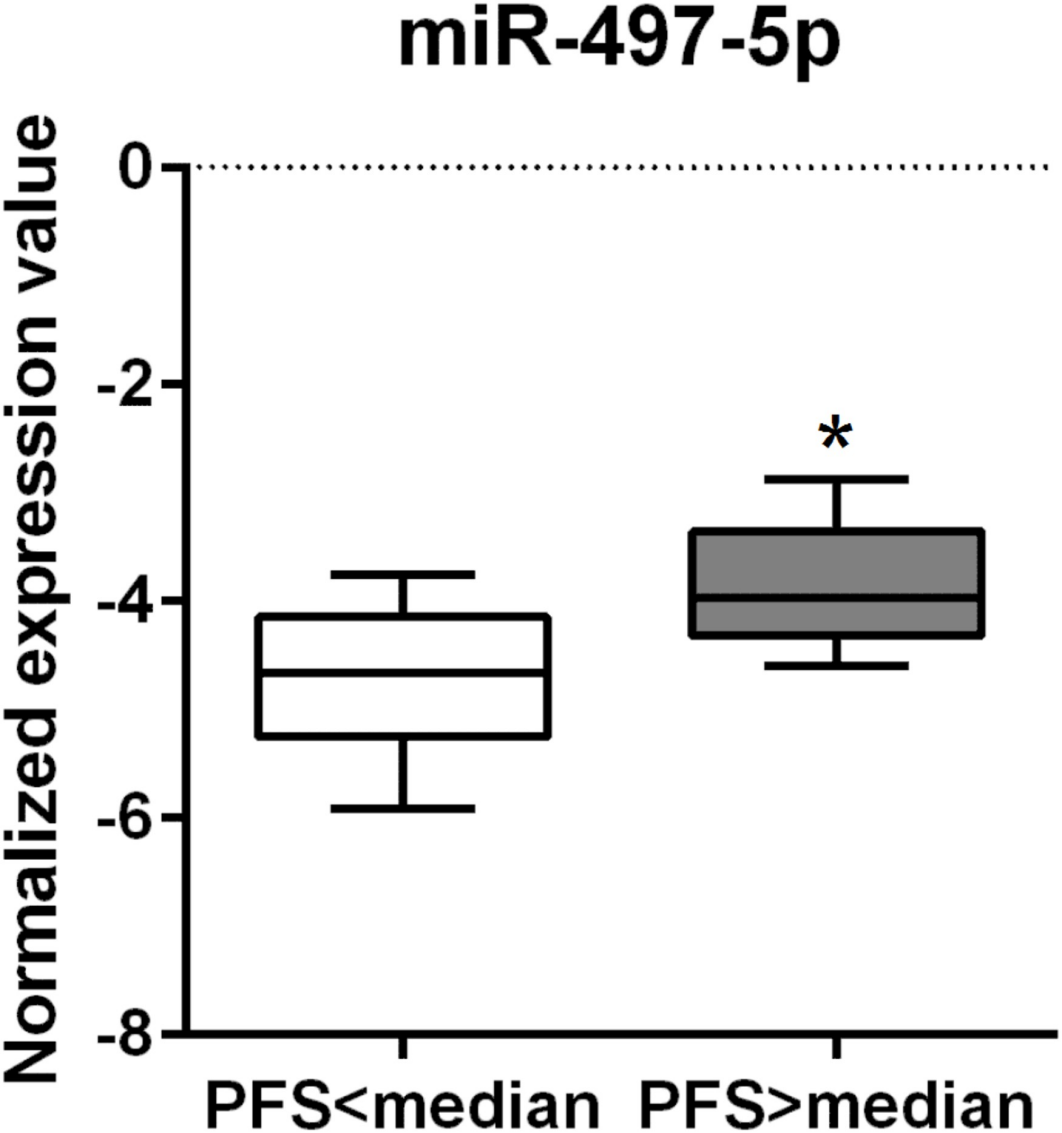
Box plot demonstrating the levels of extracellular microvesicle miR-497-5p in plasma during treatment with MAPK- inhibitors and progression-free survival (PFS) in patients with metastatic cutaneous malignant melanoma. * P < 0.01, Mann-Whitney U-test.

The EV miR-497-5p levels during treatment were significantly higher in patients with PFS above the median compared to patients with PFS below the median of 177.5 days (*P* = 0.0052) (Fig 6).

**Fig 7:**
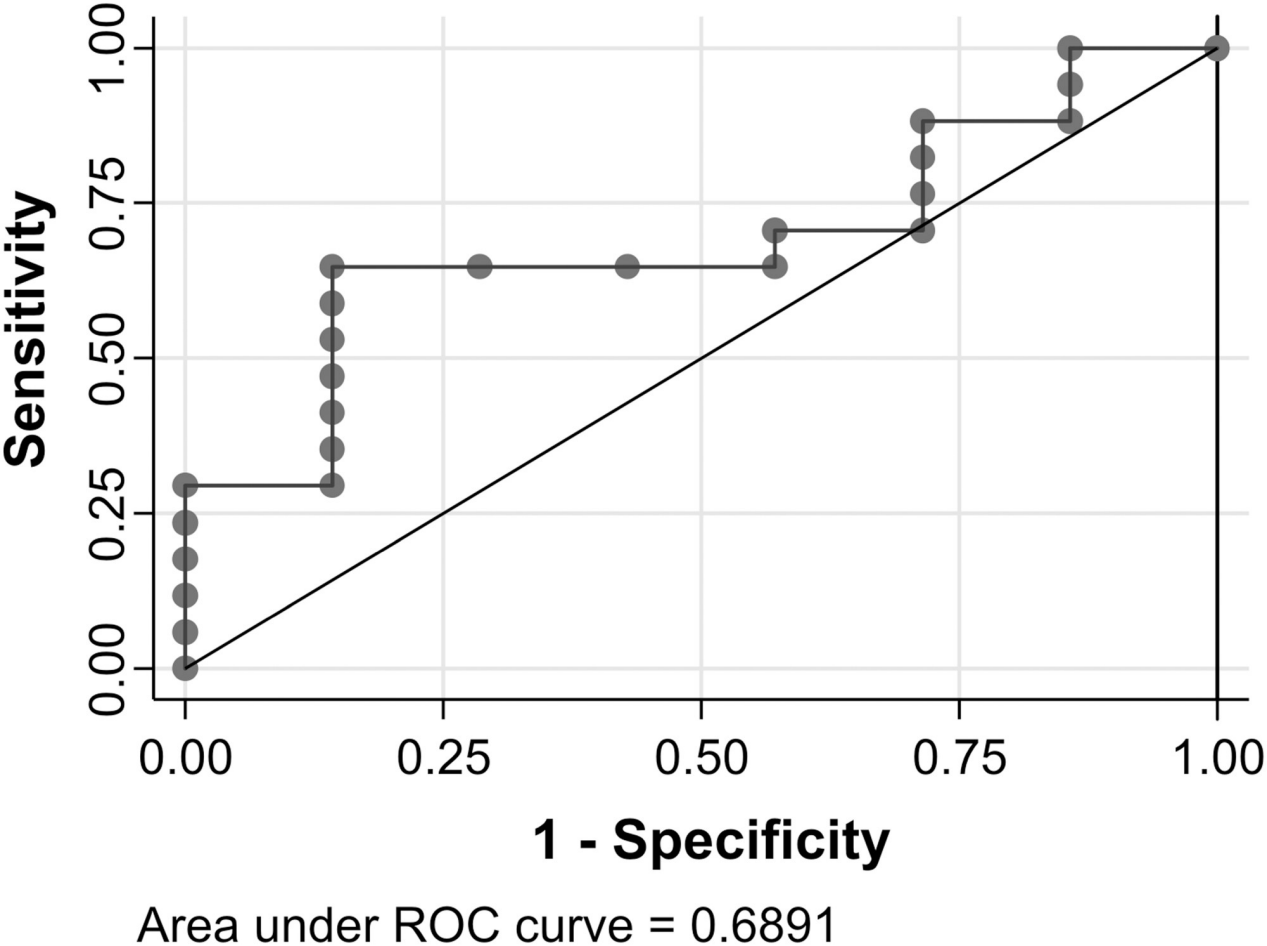
Receiver operating characteristic (ROC) curve generated from extracelluar microvesicular (EV) miR-497-5p levels in plasma and progression-free survival (PFS) of treated patients. The area under the ROC curve is 0.6891, corresponding to a poor overall accuracy of the test.

The ROC curve analysis for PFS showed that the AUC significantly separated the two groups of patients according to the median level of miR-497-5p during MAPKi treatment in relation to PFS (AUC = 0.69, P = 0.028) with a sensitivity and specificity of 64.7% and 85.7%, respectively (Fig 7).

The qPCR analysis of selected EV miRNAs in plasma samples collected before and during treatment as well as on disease progression from 8 patients showed that EV miRNA 497-5p was not detectable in five of eight samples collected during progression (S5 Table).

Full miRNA qPCR array data is deposited in NCBI’s Gene Expression Omnibus (GEO), with accession number GSE102166.

## Discussion

In the present study we assessed EV miRNA levels to identify predictive biomarkers in matched plasma samples in patients with metastatic *BRAFV600* mutated CMM receiving therapy with MAPKis. Patients with increased delta levels of EV let-7g-5p had a higher probability of DC. CMM patients with high levels of EV miR-497-5p during MAPKi therapy had a significantly longer PFS. To our knowledge, this is the first report of a significant association between EV miRNA levels and CMM treatment outcome.

Previous studies have demonstrated that miRNAs from the let-7 family regulate RAS expression in human cells, and therefore may act as tumor suppressors in several cancer forms [25–28]. In lung cancer, let-7 has been shown to be down regulated compared to expression in normal lung tissue and loss of let-7 has been correlated to a reduced postoperative survival [25]. Other data indicate an association of the let-7 family with outcome of chemo and radiotherapy. In uveal melanoma, high expression of let-7g has been associated with better outcome of chemo- or radiotherapy [27]. The up-regulation of let-7g-5p has also been correlated with improved PFS and OS in patients with ovarian cancer receiving neoadjuvant chemotherapy [28] and similar data is reported in rectal cancer for high expression of let-7g [26].

High expression of miR-497 has been shown to suppress MEK1, RAF1 and ERK1 proteins in HeLa cells, thus functioning as a tumor suppressor [29]. In resected metastases from patients with metastatic III (lymph nodes) and IV CMM, increased levels of MiR-497-5p have been associated with prolonged post-recurrence survival [30]. In addition, up-regulation of miR-497-5p has been associated with chemotherapy sensitivity in osteosarcoma cell lines, whereas data indicate that low expression may be linked to multi-drug resistance in both lung- and gastric cancer cell lines [31–32].

These previous reports provide a biological rationale supporting the correlation between increased EV levels of let7g-5p and mir-497-5p with a favorable outcome observed in our study. In addition, the frequent lack of detectable EV miRNA 497-5p in samples taken on progression strengthens our hypothesis.

Previous reports have also shown the potential role of tumor derived EVs, mainly exosomes, as biomarkers to predict CMM outcome and resistance to MAPKis [14,16, 17, 33–35]. Data also indicate that CMM released exosomes may contribute to the establishment of metastases by educating bone marrow–derived cells toward a pro-metastatic phenotype, generating pro-angiogenic events and modifying the extracellular stroma at pre-metastatic sites [33–34]. BRAFi sensitive CMM cell lines treated with vemurafenib have also been reported to secrete EVs contributing to resistance, tumor cell survival and dissemination of resistant CMM cells [14,16]. In addition, recently published study has shown that vemurafenib increased the number of total EV RNA and proteins from melanoma cells after treatment and that the EVs cargo is also affected by treatment with BRAFi [16].

In the present study, higher LDH levels before treatment were correlated with shorter PFS. This finding is consistent with a previous report where female sex and normal LDH levels were correlated with favorable PFS and OS in stage IV CMM patients treated with MAPKIs [36]. This indicates that LDH levels need to be compared to and possibly combined with novel treatment predictive biomarkers to further improve the predictive power [36].

The strength of our report is the uniqueness of the data. Although being an exploratory study, this is the first investigation to date analyzing matched plasma samples showing that EV miRNA levels are correlated with outcome in patients with *BRAFV600* mutated metastatic CMM receiving MAPKis.

The main limitation is the power of the findings considering the small sample size, and few events, in relation to the number of EV miRNAs investigated. This was mainly observed when analyzing the correlation of EV miRNAs levels with therapy response, which generated a very high OR. A validation of the results in a larger cohort should preferably include the analysis of corresponding tumor tissues to better understand the origin of the EV miRNAs since even cells in the tumor microenvironment may be affected by treatment and thus release EVs [14]. However, existing data on let7g-5p and miR-497-5p in CMM support their origin from CMM cells.

For analyzing EVs, we used a polymeric precipitation technique and low-speed ultracentrifugation to collect a precipitate that was expected to contain exosomes. This is a fast and effective technique, but non-EVs contaminants such as lipoproteins in the precipitate may occur [37]. During optimization of the methods, CD63 western blots and the electron-microscopy demonstrate that the extracts from serum samples from healthy donors did contain exosomes, but it should be emphasized that we do not claim that the miRNAs detected originate exclusively from exosomes, hence the term we use is consistently EV.

In conclusion, we showed that miRNA levels in plasma derived EVs may be predictive of treatment outcome in metastatic CMM patients receiving MAPKis. Increased levels of EV delta let-7g-5p and of miR-497-5p during treatment were identified as putative novel predictive biomarkers of MAPKi treatment benefit in this patient population. These results should be validated in a larger cohort.

## Supporting information

S1 Fig Exosome characterization. (TIF)

S1 Table Stability values of extracellular microvesicle (EV) microRNAs in all plasma samples used for data normalization and defined by the normfinder algorithm. (TIF)

S2 Table Lactate dehydrogenase (LDH) levels before and during treatment with MAPK-inhibitors in 28 patients with metastatic cutaneous malignant melanoma. (TIF)

S3 Table The correlation between extracellular microvesicle (EV) microRNA* levels in plasma samples collected before treatment, and therapy response in 26 patients with metastatic cutaneous malignant melanoma receiving MAPK-inhibitors. (TIF)

S4 Table The correlation between extracellular microvesicle (EV) microRNA* levels in plasma samples collected during treatment and therapy response in 25 patients with metastatic cutaneous malignant melanoma treated with MAPK-inhibitors. (TIF)

S5 Table Result from the qPCR analysis of let7g-5p and miR-497-5p in plasma samples before treatment (BT), during treatment (DT) and at disease progression (DP) from eight patients with metastastatic cutaneous malignant melanoma treated with MAPK-inhibitors. Cel-miR-39 was used as a spike-in control.
(TIF)

S6 Table The correlation between extracellular microvesicle (EV) microRNA* levels in plasma samples before treatment and progression free survival in plasma in 28 patients with metastatic cutaneous malignant melanoma treated with MAPK-inhibitors. (TIF)

S7 Table The correlation between extracellular microvesicle (EV) microRNA* delta_levels** in plasma samples and progression free survival in 25 patients with metastatic cutaneous malignant melanoma receiving MAPK-inhibitors. (TIF)
