# Supplementary figures and images for: Extracellular microvesicle microRNAs as predictive biomarkers for targeted therapy in metastastic cutaneous malignant melanoma
Source: PLoS One. 2018 Nov 6;13(11):e0206942. doi: 10.1371/journal.pone.0206942 (PMC6219796; doi:10.1371/journal.pone.0206942)

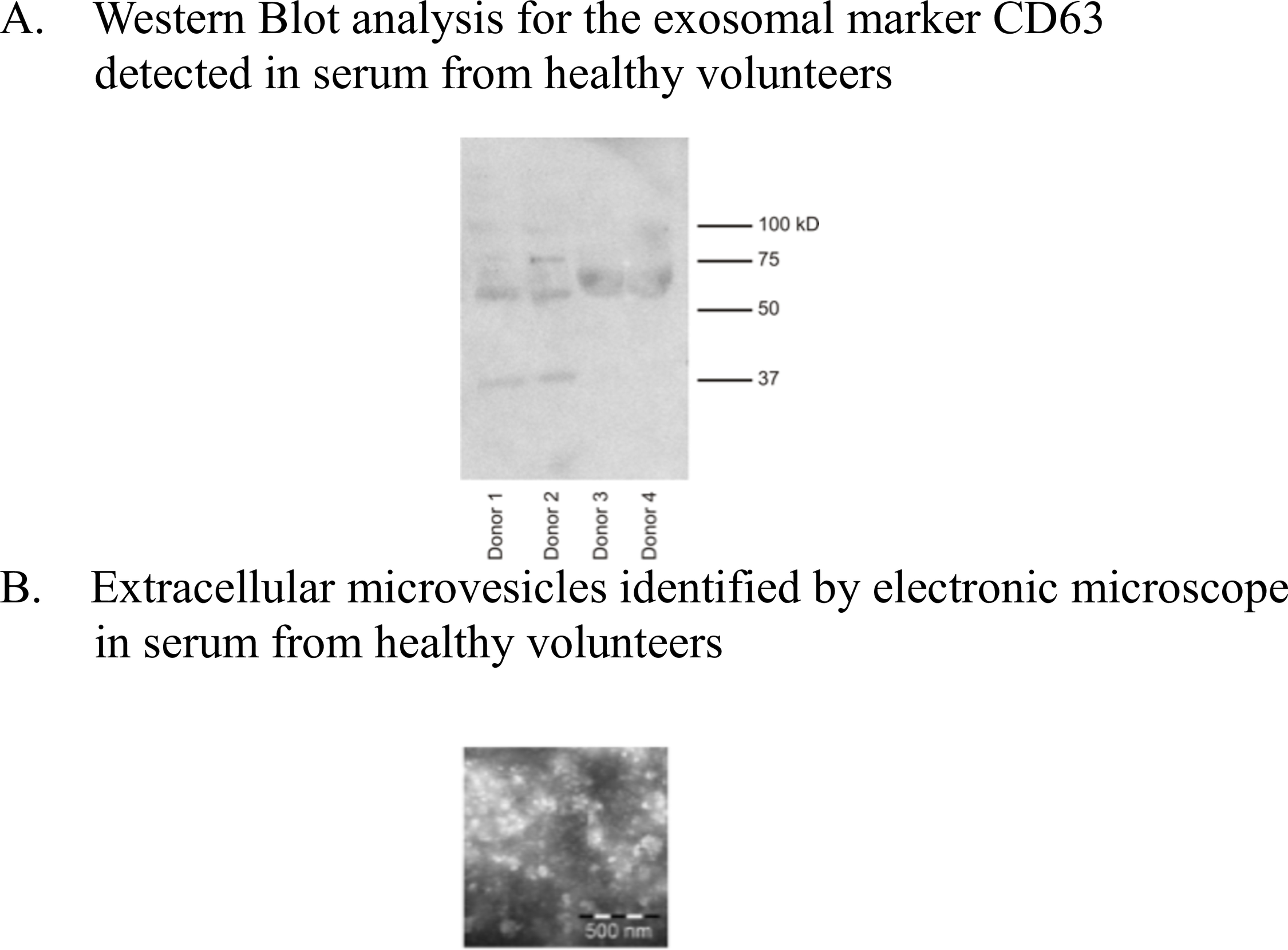

Supplement: S1 Fig — (TIF) [file pone.0206942.s001.tif]

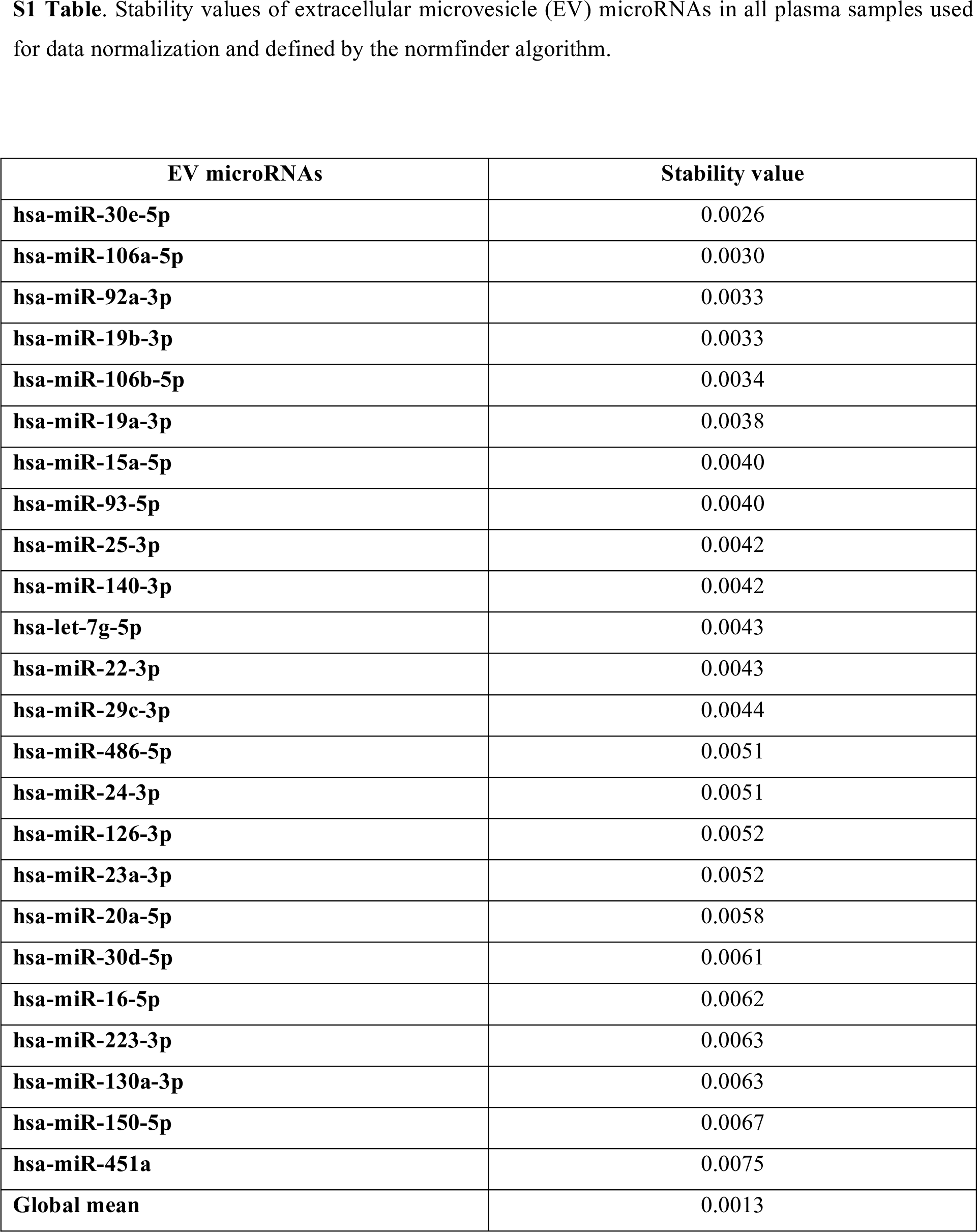

Supplement: S1 Table — (TIF) [file pone.0206942.s002.tif]

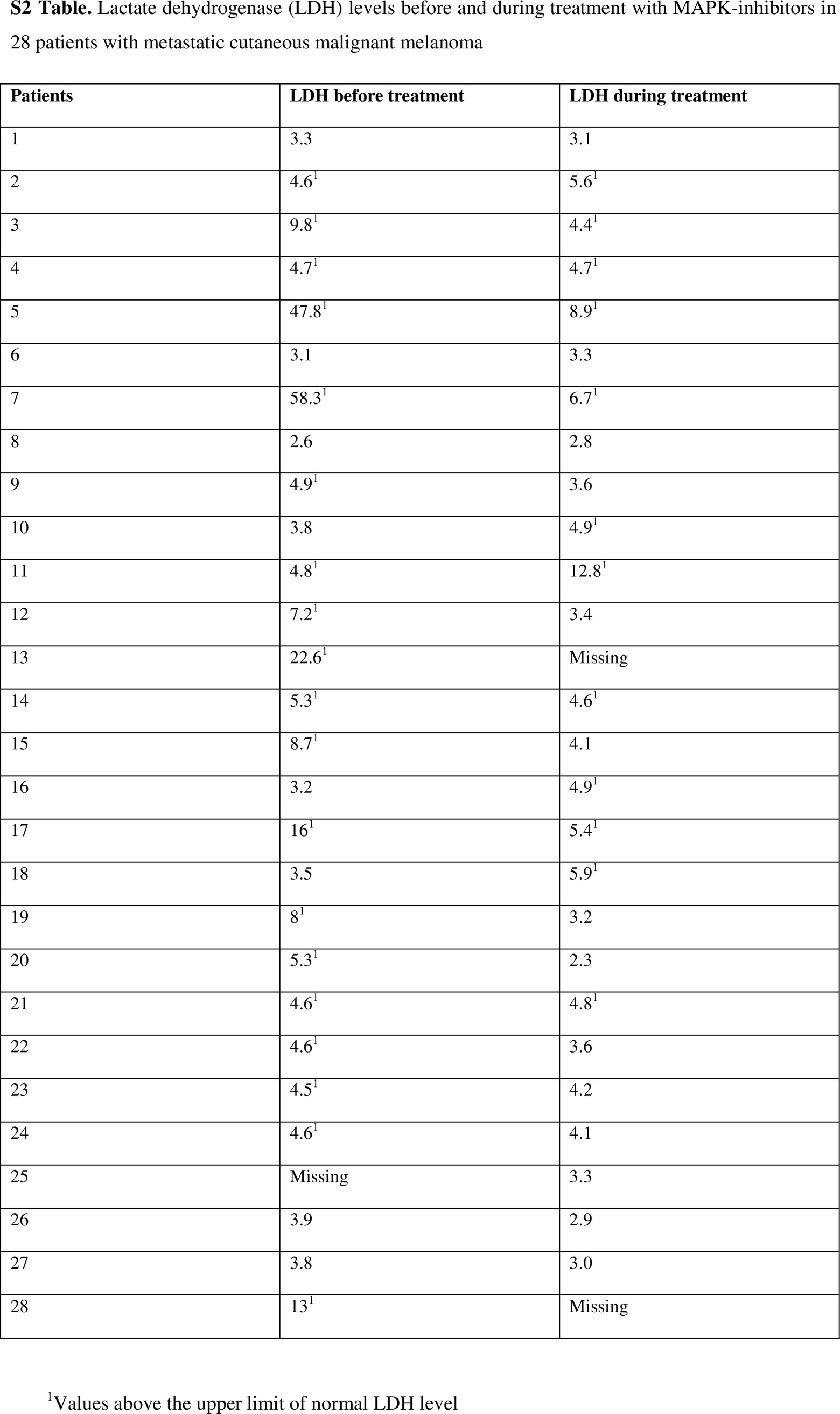

Supplement: S2 Table — (TIF) [file pone.0206942.s003.tif]

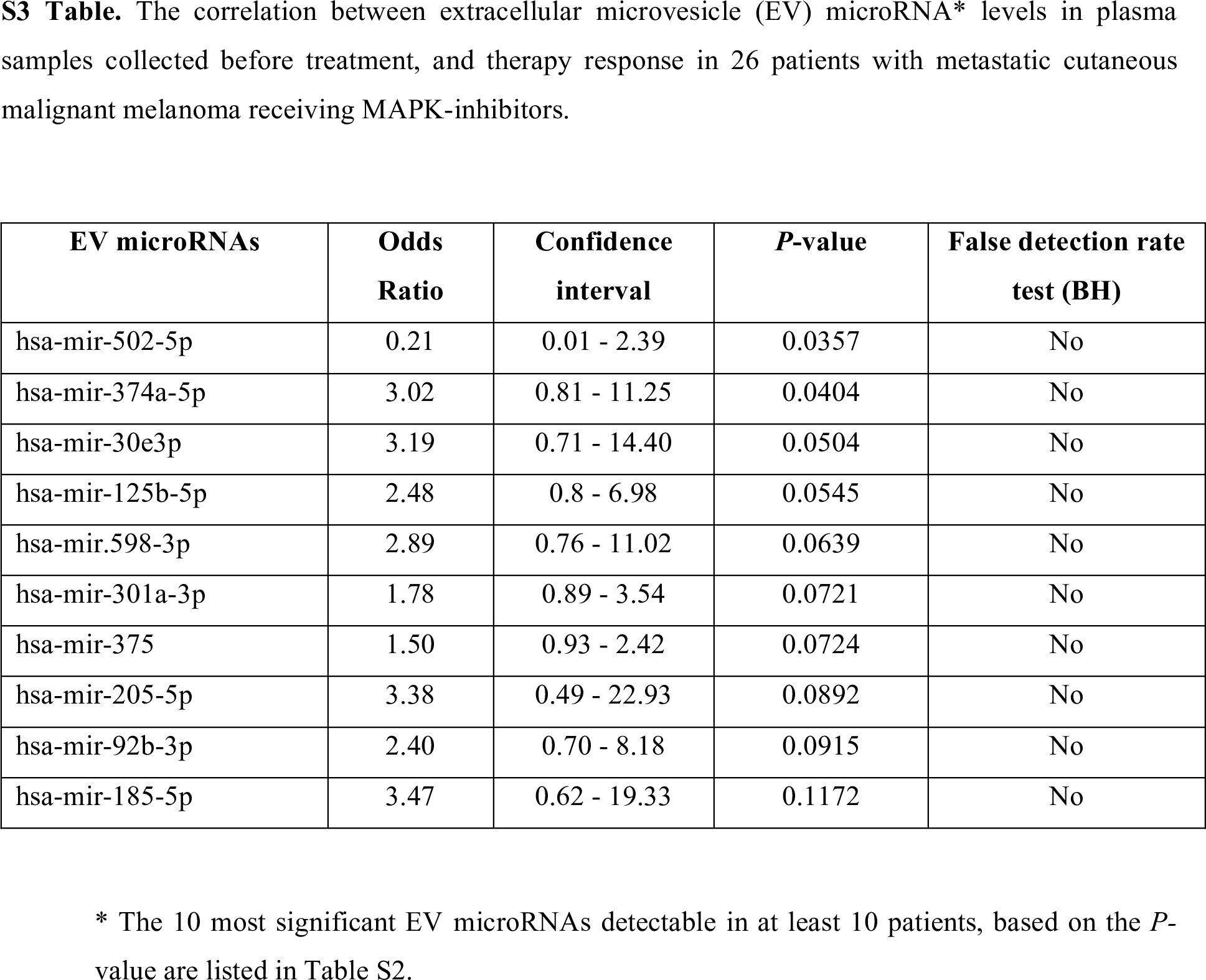

Supplement: S3 Table — (TIF) [file pone.0206942.s004.tif]

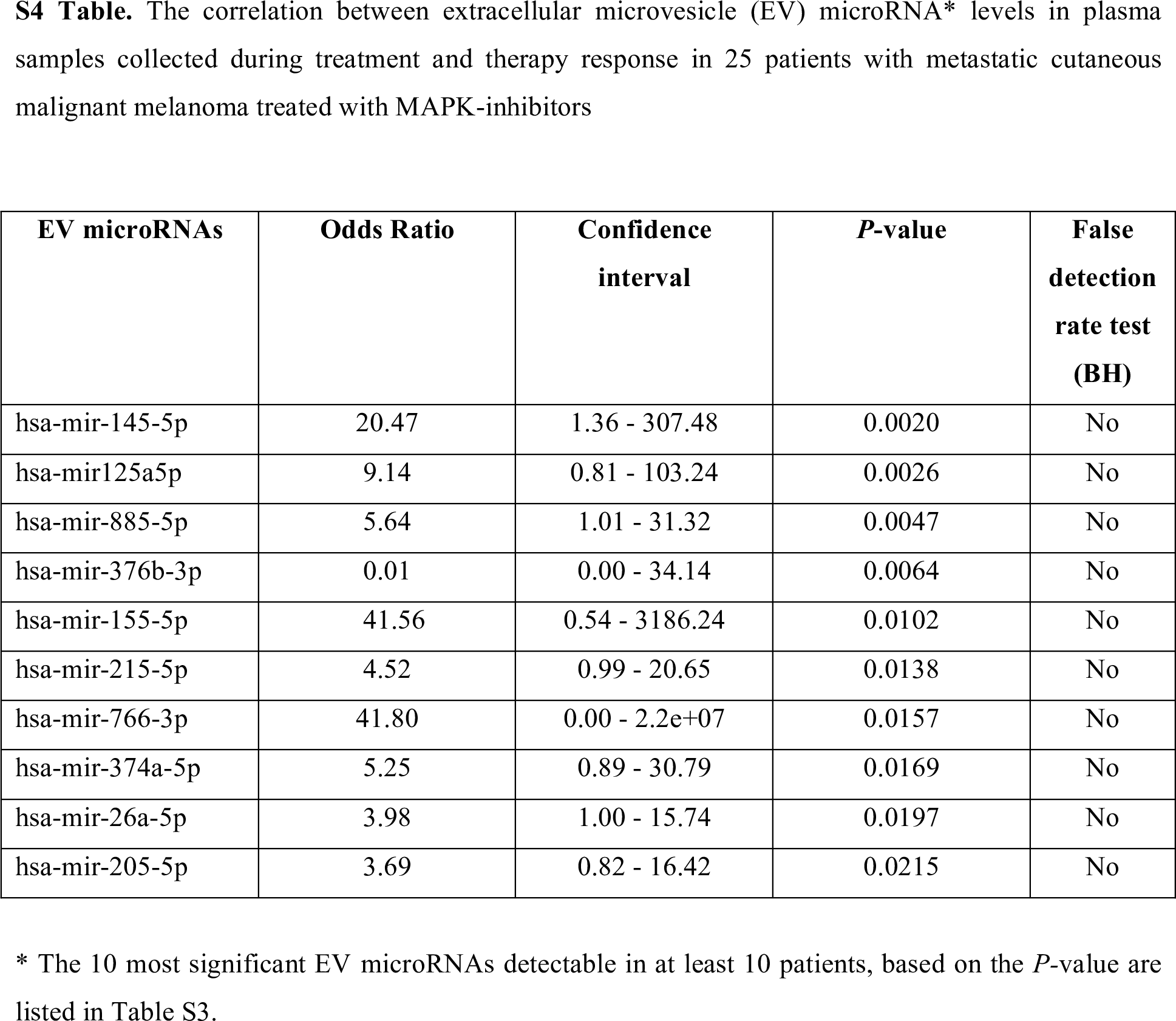

Supplement: S4 Table — (TIF) [file pone.0206942.s005.tif]

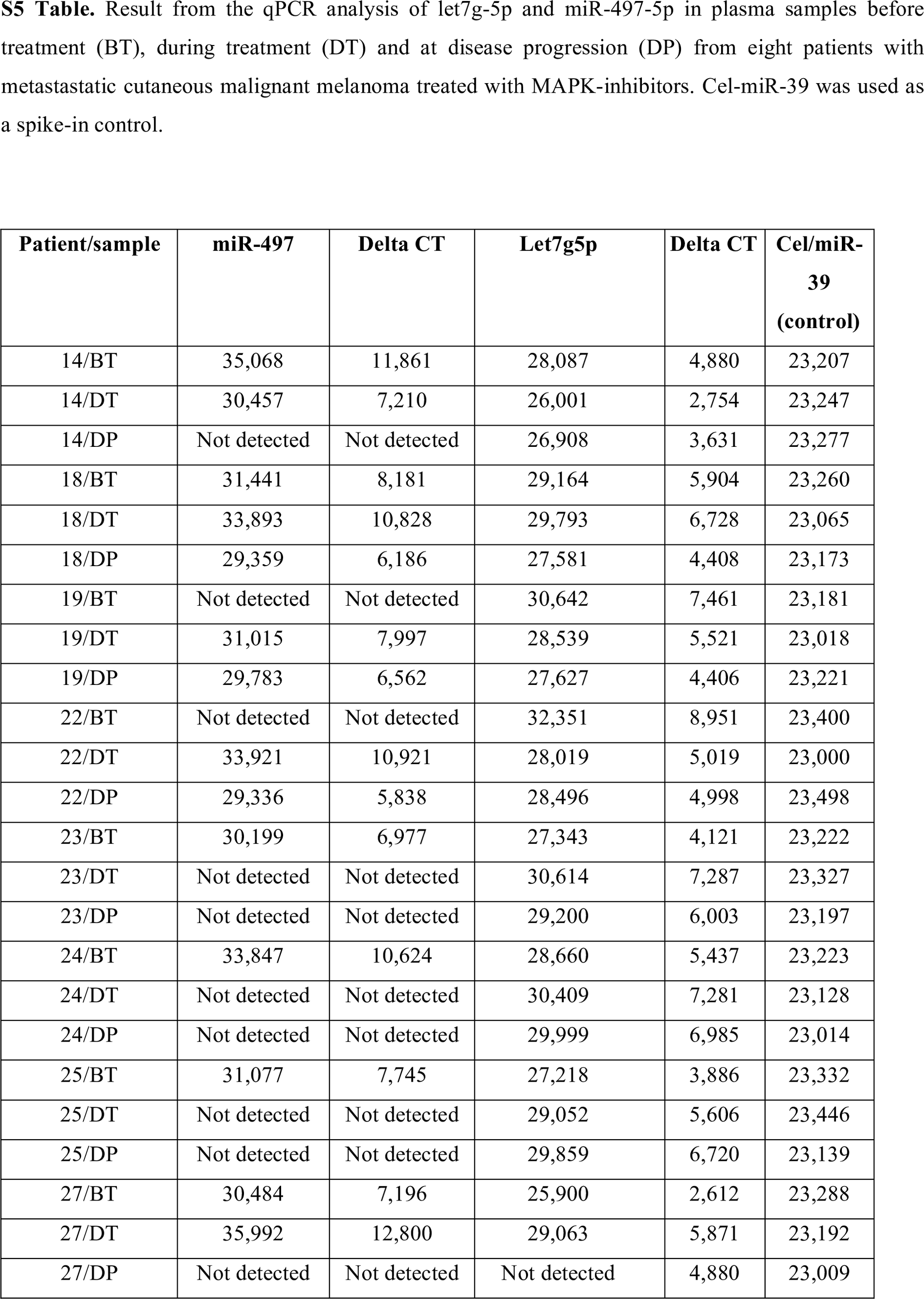

Supplement: S5 Table — Cel-miR-39 was used as a spike-in control. (TIF) [file pone.0206942.s006.tif]

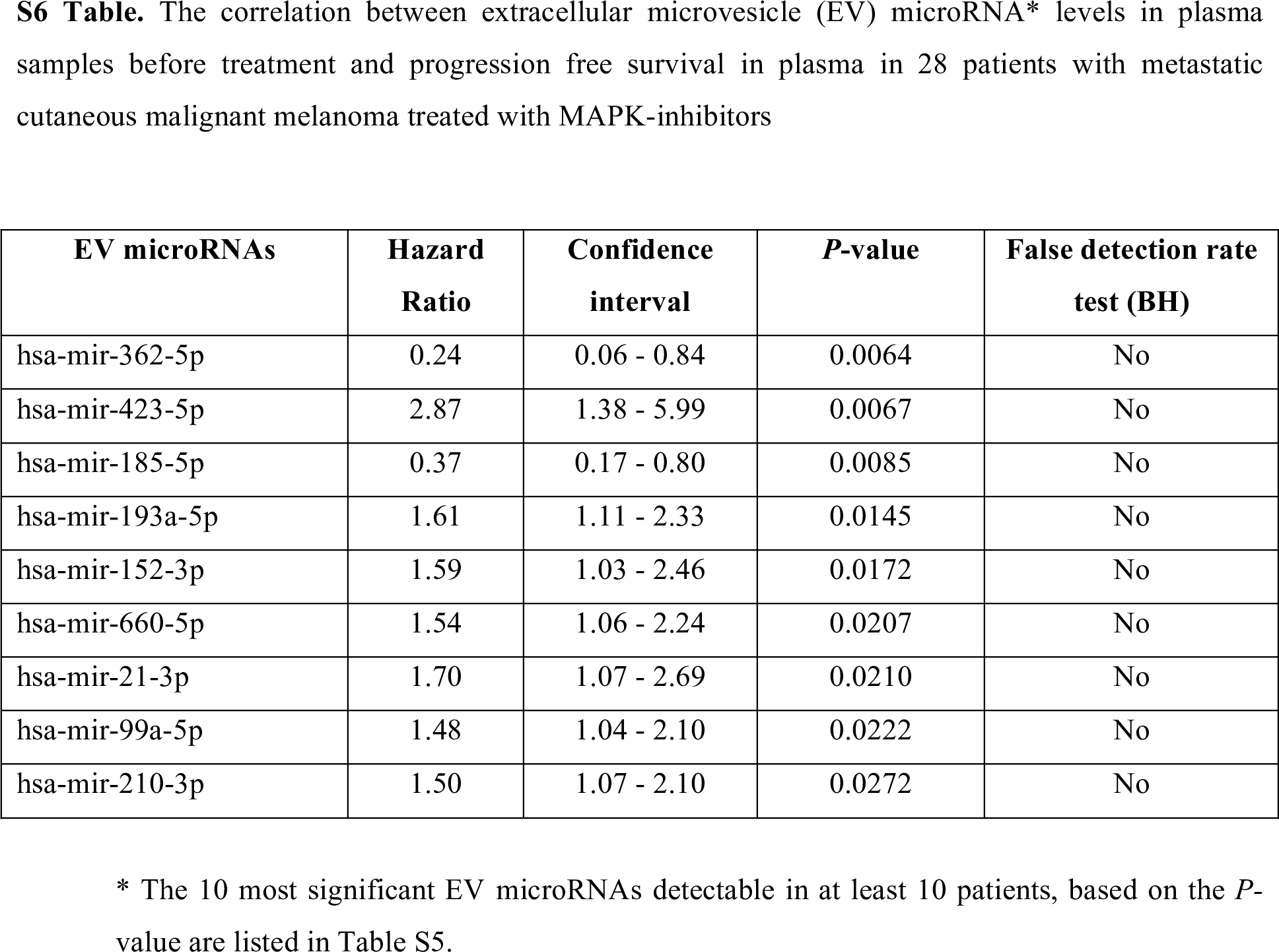

Supplement: S6 Table — (TIF) [file pone.0206942.s007.tif]

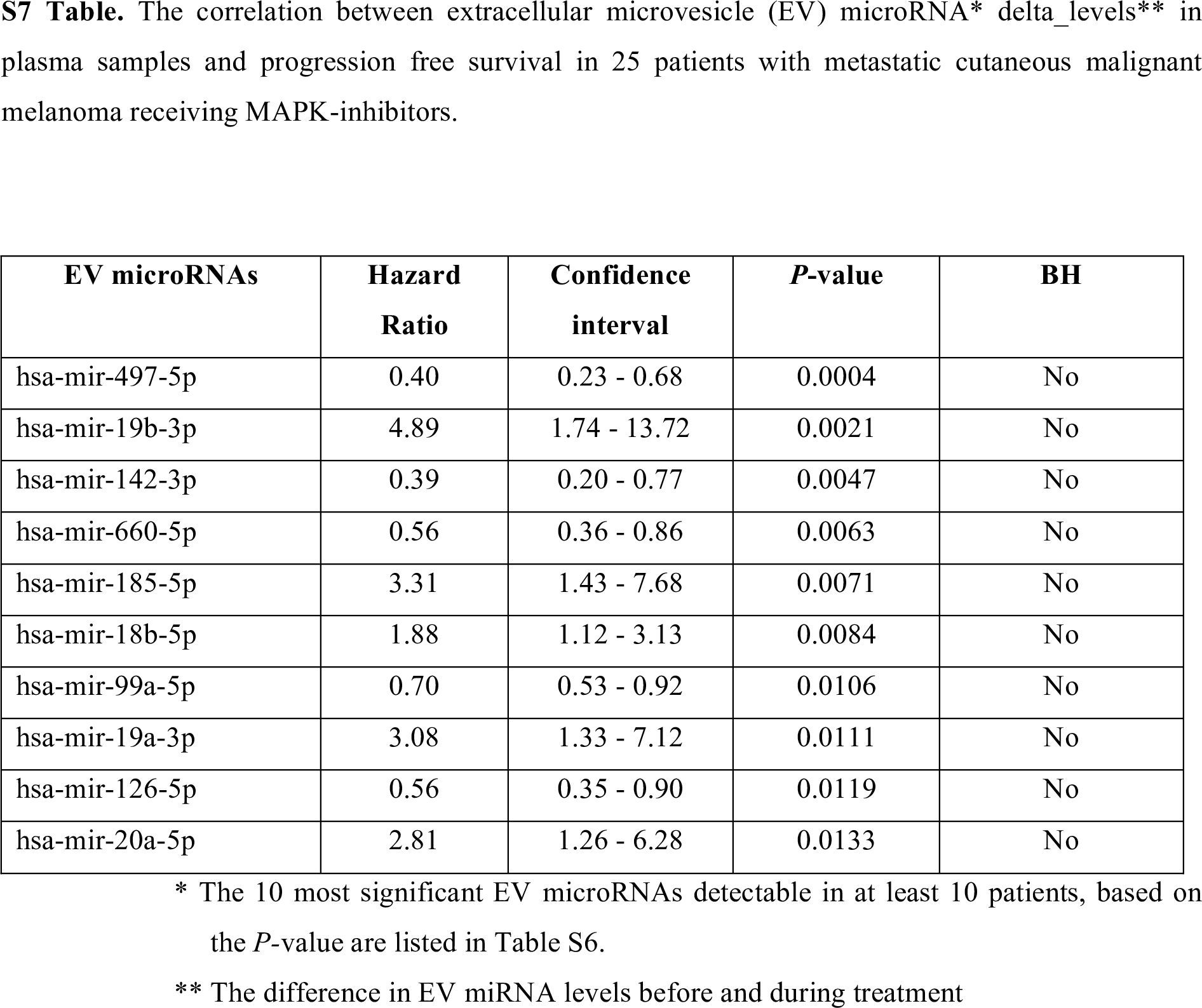

Supplement: S7 Table — (TIF) [file pone.0206942.s008.tif]
